# 

**Published:** 1874-10

**Authors:** 


### Contents of October Number.

### ORIGINAL DEPARTMENT.

ART. I.—Syphilitic Affections of the Nervous System. By Edward N. Brush, M. D. .......... 81

ART. II.—Proceedings of Albany County Medical Society. Reported by F. C. Curtis, M. D., Secretary .......... 92

ART. III.—Abstract of Proceedings of the Buffalo Medical Association. Reported by E. R. Barnes, M. D., Secretary, .......... 106

ART. IV.—Clinical Remarks on Surgical Cases occurring at the Buffalo Hospital of the Sisters of Charity, by Prof. J. F. Miner, M. D. Reported by W. W. Miner, M. D. .......... 113

### EDITORIAL DEPARTMENT.

The Recent Grave Robbery in this City, .......... 117

Correspondence, .......... 118

Notice to Subscribers, .......... 119

BOOKS REVIEWED :

A Hand-book of Obstetric Surgery. By Chas. Clay, M. D., etc. 119

Books and Pamphlets Received .......... 120

